# The Utility of Pulse Fluoroscopy During Mediport Insertion to Diagnose Air Embolism

**DOI:** 10.5152/TJAR.2021.21113

**Published:** 2022-06-01

**Authors:** Mihika Shah, Enrique J. Pantin

**Affiliations:** 1 Rutgers Robert Wood Johnson Medical School, Rutgers University, New Brunswick, USA; 2 Department of Anaesthesiology and Perioperative Medicine, Rutgers Robert Wood Johnson Medical School, New Brunswick, USA

**Keywords:** Air embolism, air embolism detection, central line complication, embolism

## Abstract

An otherwise healthy man in his 40s recently diagnosed with esophageal adenocarcinoma sustained an air embolism during the insertion of a mediport under mild sedation that was noted while using pulse fluoroscopy to ensure good visibility of adequate placement of the catheter tip. Pulse fluoroscopy allowed the early detection of a potentially catastrophic situation caused by air in the right heart and main pulmonary artery, thus allowing prompt correction of the mistake that had allowed the air embolism to occur. Pulse fluoroscopy eliminates or greatly reduces the blurred vision of highly mobile objects and enhances the view of low contrast objects thus enhancing imaging quality.

**Main Points**

- Tools, such as a checklist, second pair of eyes from a nondirect procedural participant, and so on, to reduce risk during critical procedural steps should be considered for all procedures with a risk of deadly complications.
- Early detection of air embolism could be enhanced by using fluoroscopy during vascular access and catheter insertion instead of still X-ray images.
- Observing what the surgical team is doing during critical portions of the case could prevent or allow early detection of potential complications.

## Introduction

Massive air embolism is a relatively uncommon complication but when it presents, it can cause sudden cardiovascular collapse and/or acute brain dysfunction with potentially devastating consequences including brain death or death. Most of the devastating massive air embolisms that we see in clinical practice are either iatrogenic or related to major trauma. The removal of long-lasting central vascular access with the enduing endothelialization of the catheter and an open skin to intravascular path is well described as a cause of sudden cardiovascular collapse postcentral line removal. Open vascular or lung injuries, open catheters to the atmosphere with passive or active air instillation, open heart surgery, and intracardiac defects with right to left communications are associated with intravenous air entraining and are among the most common causes of air-related cardiovascular collapse. Due to the relatively low frequency of these events, clinicians do not actively think of them during a major unexpected cardiovascular collapse. This case demonstrates how a simple mistake – lack of clamping a central line catheter– had the potential of causing a major cardiovascular event, and how a vigilant anaesthesia provider was able to prevent a potentially deadly complication. Written informed consent was obtained from the patient to publish his case.

## Case Presentation

A 42-year-old male, 178 cm tall, and weighing 78 kg was recently diagnosed with poorly differentiated adenocarcinoma of the esophagus. He required systemic chemotherapy and was brought into the operating room for a mediport (Deltec®, Port-A-Cath®, 1.6 mm internal diameter) insertion. The patient was connected to standard monitoring (blood pressure, EKG, and SpO_2_) and an oxygen cannula with EtCO2 monitoring capability placed at 3 L min^-1^ of oxygen. Mild sedation with midazolam 2 mg intravenously was provided. After being prepped, draped in a sterile fashion, and placed in the Trendelenburg position, local anaesthesia with 2% lidocaine was administered by the surgeon. The right internal jugular vein was cannulated with the aid of ultrasound, and a wire was passed into the vein. Fluoroscopy was used to confirm the placement of the wire within the central venous system. A pocket was made in subcutaneous tissue for port placement, and a catheter was tunneled from the chest site to the port insertion site in the neck and brought out through the skin. A peel-away catheter was mounted over the wire and introduced into the jugular vein. The introducer dilator was removed, and the port catheter was inserted through it inside the jugular vein. At this point, the bed was placed in the neutral position, and pulsed fluoroscopy was used to ensure adequate placement of the catheter tip at the junction of the superior vena cava (SVC) and the right atrium (RA). Pulse fluoroscopy^1^ allowed a short dynamic view of the catheter tip as it moved with the cardiac cycle which is ideal when imaging low contrast objects and structures. This technique eliminates or greatly reduces the blurred vision of highly mobile objects—in this case, the proximal tip of the mediport catheter due to cardiac activity.

When examining the fluoroscopic loop, the anaesthesiologist noted a mobile abnormal shadowy image ill-defined in the RA, right ventricle (RV), and more noticeable in the area just past the pulmonic valve (PV) and warned the surgical team that air was being entrained. This abnormal image was obvious on the dynamic loop but not on the still image, as its visualization had been enabled by using pulsed fluoroscopy as opposed to a single shot X-ray exposure. The patient remained asymptomatic with stable vital signs at this time.

The sudden ability to obtain a view of the right heart, even though the image was ill-defined and blurred, raised the immediate question of “what is making this possible?”

**Figure 1.**
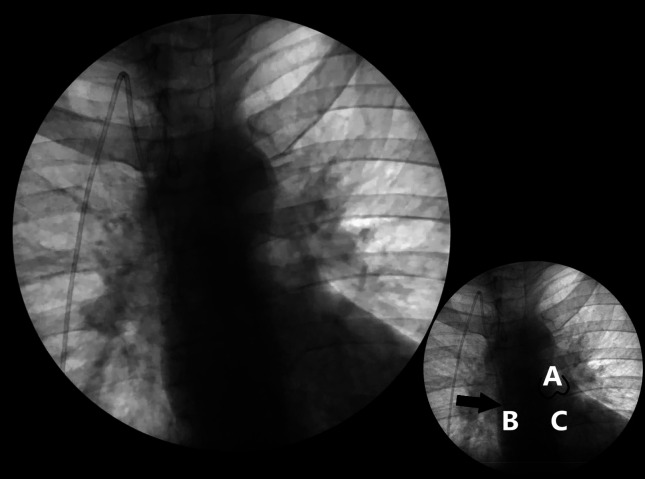
Note Mediport catheter distal catheter tip inside the superior vena cava. In the area of the right ventricle, but specifically above the pulmonic valve, note a degree of radiolucency not present in the rest of the cardiac silhouette which is compatible with intracardiac air. This effect is clearly seen in the video loop obtained during the case. Mediport insertion with proximal catheter open to air and distal catheter tip (black arrow) inside the superior vena cava. Letters over cardiac silhouette generated by the air are: pulmonic valve (A), right atrium (B), and right ventricle (C). Video: https://youtu.be/DwEeQ9YPVnI

It was noted that the proximal portion of the port catheter, which was laid over the patient’s draped chest, had not been clamped after the distal tip had been inserted into the central venous system and it was open to the air. This served as the source of the air embolism that served as a contrast agent inside the heart (Figure 1). The proximal catheter was clamped once this was observed, and the patient was placed into the Trendelenburg position.

The fluoroscopic loop showed a systolic and diastolic “blurred” view of the RA and ventricle and a clear view of the PV due to the intracardiac air as seen in the video provided. Due to the rapid diagnosis, the patient had no postoperative complications.

## Discussion

Air is a natural radiographic contrast, useful in chest and gastrointestinal studies, that does not cause opacification but instead adds transparency to radiological images. This case illustrates how even during common procedures the lack of adherence to proper technique can lead to catastrophic complications. In this case, proximal catheter clamping or connecting the proximal catheter with the port reservoir before inserting the catheter through the sheath would have eliminated the risk of a massive air embolism. The lack of proper technique was noted early by the anaesthesia provider looking at the fluoroscopic imaging during a mediport insertion. Due to the patient’s hemodynamic stability and to prevent further patient motion (which could have potentially dislodged the air accumulated in the right heart), it was decided not to transport the patient to the computed tomography suite for further study but to manage conservatively with prolonged observation and further surface ultrasound assessment done without patient position changes. The decision to add an iodine contrast agent to better visualize the cardiac and pulmonary artery silhouette was also decided against as this would not have improved patient care.

There are only a few agents that have been used as contrast material to enhance the visualization of structures during radiological tests. Currently, only intravenous iodine agents are used as contrast material. Previously, air was used for brain imaging, contrast-like air/powdered diatrizoic acid was used for tracheo-bronchography,^2^ and air/barium was used for colonic double-contrast studies. Air and non-iodine agents are no longer used as contrast agents for radiological studies as newer technology has long replaced their need. Other gases such as CO_2_ are being studied as alternative intravascular agents for patients with iodine contrast allergy or compromised renal function.^3^

Although iatrogenic air embolism is a relatively rare but potentially life-threatening complication, care must be taken to avoid leaving intravascular ports open to the atmosphere which creates conditions to allow air to be injected or introduced into the patient.^4^ Air can also be introduced to the vascular system due to traumatic events that create pathways between the vascular system and the airway or lungs.^5^ Additionally, patient positioning to increase venous pressure, preventing hypovolemia, and introducing the catheter during expiration when the negative intrathoracic pressure is at its minimum should be considered to decrease the risk of air embolism.^6^ If venous embolism does occur, some maneuvers to consider include: placing the patient in the left lateral decubitus and/or Trendelenburg positions, trying to suction the intracardiac air with intracardiac catheters, or placing the patient in an oxygen-rich environment.^7^

Paying attention to the bed position and potential sources of air entrapment during central vascular access and confirming that the catheter lumen remains closed are paramount during central line insertion and manipulation. The addition of a separate “time out” to emphasize the precautions and maneuvers necessary to prevent air embolism or bleeding during central access should also be considered. Also, when available, the use of pulsed fluoroscopy is a useful technique compared to a single shot X-ray exposure. When air embolism has already occurred, there are considerations based on the clinical scenario, location of the air, and amount and potential for limiting its transit into the pulmonary circulation that are beyond the scope of this case report. This is a unique case of pulse fluoroscopy utilization during a mediport insertion, which allowed better visualization of an air embolism in the right heart, specifically the supra-PV region that ultimately served as an added monitoring tool that prevented a potential adverse outcome. Fluoroscopy is used infrequently by anaesthesia providers for intravascular lines as proper use of ultrasound has become the primary tool used. In certain scenarios, such as placing lines where the whole path of a vascular wire or catheter needs to be followed through the vascular system, ultrasound combined with fluoroscopy is required for maximum safety and effectiveness.

## Conclusion

This case emphasizes the need for constant vigilance during every case, not only on your scope of practice but holistically as this enhances the early detection of a potentially deadly complication.
